# Coexisting Pulmonary Tuberculosis and Mucormycosis in a Patient with Aplastic Anemia Post Allogenic Stem Cell Transplantation

**DOI:** 10.4084/MJHID.2011.0036

**Published:** 2011-09-08

**Authors:** Sanjeev Kumar Sharma, Narendra Agarwal, Anjan Mukherjee, Tulika Seth, Pravas Mishra, Immaculata Xess, Manoranjan Mahapatra, Sanjay Sharma

**Affiliations:** 1 Department of Hematology; 2 Microbiology and; 3 Radiodiagnosis, All India Institute of Medical Sciences, New Delhi, India

## Abstract

Infections are the most common cause of morbidity and mortality in allogenic stem cell transplant recipients. Survival of the patient depends on the accurate diagnosis of the infectious agents and prompt and effective management of the infection alongwith maintenance of adequate immunosuppression post transplantation. We here reported a case of aplastic anemia who developed left upper lobe consolidation post allogenic stem cell transplantation and was found to have combined infection with tuberculosis and mucormycosis. This is the first case of combined infection with tuberculosis and mucormycosis reported in such a host, with a favourable outcome

Mucormycosis is a dreaded complication in immunocompromised patients particularly following chemotherapy or stem cell transplantation, and can be highly fatal if not treated urgently.^1^ Tuberculosis, which is particularly common in developing countries, is now being increasingly recognised in transplant recipients, can pose both diagnostic and therapeutic challenges in such patients.^2^ We report a case of aplastic anemia who developed left upper lobe consolidation post allogenic stem cell transplantation and was found to have combined infection with tuberculosis and mucormycosis. Although isolated infections by tuberculosis and mucormycosis have been reported in transplant recipients, combined infection with both simultaneously in transplant recipients has not yet been reported.

A 26-years old lady presented with progressive weakness and fever for 3 months, and was found to have pancytopenia (hemoglobin 6.5g/dl, total leukocyte count 2.8x10^9^/l, neutrophils 12% and lymphocytes 88% and platelet counts 13x10^9^/l). Bone marrow biopsy revealed cellularity of 5%. She was diagnosed severe aplastic anemia and had HLA matched sibling. She underwent allogenic peripheral blood stem cell transplantation (PBSCT) from the matched sibling donor, with conditioning regimen including fludarabine 30mg/m^2^ for 6 days (from day −10 to day −5) and cyclophosphamide 60mg/m^2^ for 2 days (day −6 and day −5). The CD34+ cell dose given was 4.8x10^6^/kg. Graft versus host disease (GVHD) prophylaxis included horse anti-thymocyte globulin (ATGAM^®^ Pfizer) 30mg/kg/day for 4 days (day −4 to day −1), methotrexate (10mg/m^2^) on day+1, +3 and +6, and cyclosporine 100mg twice daily intravenously from day −1 with dose adjusted according to plasma cyclosporine levels (between 150–300ng/ml). She was given oral levofloxacin 500mg daily, fluconazole 400mg daily, aciclovir 400mg twice daily and trimethoprim-sulfamethoxazole 480 mg q12 hours on alternate days as prophylaxis. She developed fever on day +4 of transplant and was started on piperacillin plus tazobactum (4.5g i.v. q 6 hours) and amikacin (750mg i.v. q 24 hours). Chest radiograph was normal, and blood and urine cultures were sterile. She became afebrile on day +7. She engrafted on day +10 (absolute neutrophil count >0.5x10^9^/l and unsupported platelet count > 20x10^9^/l). On day + 17 she developed fever with dry cough and left sided chest pain. Computerized tomography (CT) scan of the chest showed left upper lobe consolidation with surrounding ground glass opacity. (Figure 1)

Oral voriconazole (400mg twice daily on the first day followed by 200mg twice daily) was emperically started and fluconazole was stopped. Bronchoalveolar lavage showed the presence of acid fast bacilli suggestive of mycobacterium tubercular infection (Figure 2). On day +19 she was started on anti-tubercular therapy (ATT) (isoniazid 300mg, rifampicin 450mg, ethambutol 1000mg and pyrizinamide 1500mg daily).

Considering high risk of fungal infection and interactions of voriconazole with anti-tubercular drugs, lung biopsy was performed 3 days later, which revealed broad aseptate hyphae branching at a right angle suggestive of mucormycosis (Figure 3). She was started on liposomal amphotericin B (3mg/kg/day) and voriconazole was stopped. With continued liposomal amphotericin B along with anti-tubercular drugs, the lesion started decreasing in size, and surgical resection was deferred. Patient became afebrile 2 weeks latter and follow-up CT scan and sequential chest radiographs showed a significant reduction in the size of the lesion.

On day +28 of transplant, patient developed renal dysfunction (serum creatinine 1.8mg/dl) and peripheral smear showed schistocytes. Cyclosporine was stopped in view of drug induced microangiopathy and mycophenolate mofetil 1000mg twice daily was started which she tolerated well.

She was continued on liposomal amphotericin B for 8 weeks and had nearly complete resolution of lung lesion. ATT was stopped 6 months later. Mycophenolate mofetil was stopped 18 months post transplant. She did not develop acute GVHD but had onset of limited chronic skin GVHD in 7^th^ month which was controlled with steroids (prednisolone 1mg/kg/day for 4 weeks and then in tapering doses for next 2 months), without reactivation of tuberculosis or mucormycosis. Patient is now 2 years post stem cell transplant with hemoglobin 12.5g/dl, total leukocyte count 8.6x10^9^/l and platelet counts 192x10^9^/l, with no sequele related to chest infection or GVHD.

Mucormycosis is rare in allogenic transplant recipients with an incidence of 1.9% reported in a series of 263 patients.^1^ The incidence of mycobacterial infection among stem cell transplant recipients has been estimated to be 0.6%–9.7% in United States^2^ but the incidence in developing countries may be even higher where tuberculosis is endemic.^3^

Our case highlights certain important facts. First, a high index of suspicion of tuberculosis should be kept in patients with upper lobe consolidations. Second, invasive fungal infections, particularly aspergillosis and rarely mucormycosis may occur in post transplant immunocompromised state and distinction between the two is very important as treatment differs. Voriconazole is the treatment of choice for aspergillus but is ineffective against mucormycosis which responds to amphotericin and may even require surgical debridement. Posaconazole has also been shown to be effective against mucormycosis. Thirdly, drug-to-drug interactions are very common when antitubercular drugs (e.g. rifampicin) are being used with antifungal (voriconazole) and immunosuppressive drugs (cyclosporine). Infection has to be treated adequately and simultaneously immunosuppression has to be maintained to prevent graft rejection. Bronchoalveolar lavage and lung biopsy both helped in detecting the causative organisms in our case leading to appropriate treatment. Both tuberculosis^4^ and mucormycosis^5^ have been reported as late complications following transplantation but our patient developed these infections early after transplantation. Rifampicin increases the metabolism of cyclosporine and so dose of latter has to be modified according to plasma levels. Toxicity of multiple drugs, particularly affecting renal and hepatic functions, can occur and requires careful monitoring of these organs. With increasing number of patients undergoing transplantations in developing countries, the endemic diseases also need to be considered while looking for the usual infections in immunocompromised post transplant recipients and mixed infections are a possibility. Diagnosis of mixed infections is challenging and requires a high index of suspicion and adequate management for better outcome, particularly when there is an emerging trend of fungal infections in patients with pulmonary tuberculosis.^6^

## Figures and Tables

**Figure 1:**
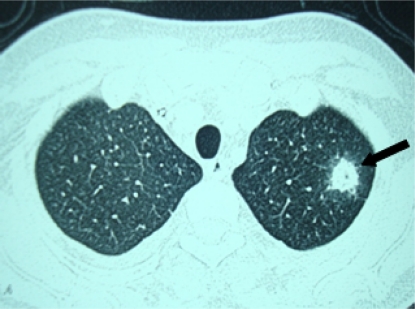
Axial CT chest showing left upper lobe consolidation with surrounding ground glass opacity (shown by arrow).

**Figure 2:**
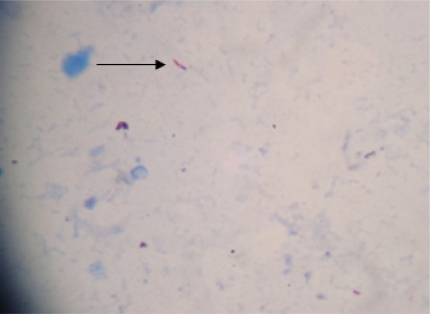
Acid Fast Bacilli in smear stained by ZN stain (shown by arrow) prepared from BAL sample of the patient after concentration and decontamination (X1000 under oil immersion).

**Figure 3:**
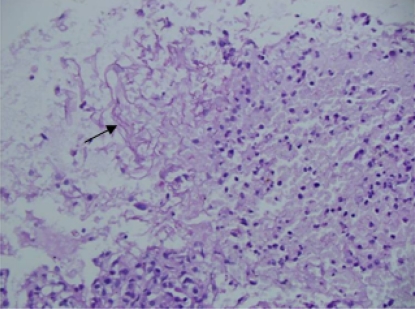
Aseptate, irregular, ribbon-like, broad, wide-angled branching hyphae suggestive of mucormycosis in lung tissue (shown by arrow) (H & E X200).
